# Mitochondrial stress response gene *Clpp* deficiency impairs oocyte competence and deteriorate cyclophosphamide-induced ovarian damage in young mice

**DOI:** 10.3389/fendo.2023.1122012

**Published:** 2023-03-24

**Authors:** Guangxin Li, Jingkai Gu, Xiaomei Zhou, Ting Wu, Xian Li, Renwu Hua, Zhuo Hai, Yuan Xiao, Jiaping Su, Willian S. B. Yeung, Kui Liu, Chenxi Guo, Tianren Wang

**Affiliations:** ^1^ Department of Breast and Thyroid Surgery, Peking University Shenzhen Hospital, Shenzhen, China; ^2^ Shenzhen Key Laboratory of Fertility Regulation, Reproductive Medicine Center, The University of Hong Kong-Shenzhen Hospital, Shenzhen, China; ^3^ Department of Obstetrics and Gynaecology, The University of Hong Kong - Shenzhen Hospital, Shenzhen, China; ^4^ Department of Obstetrics and Gynaecology, Li Ka Shing Faculty of Medicine, The University of HongKong, Hong Kong, Hong Kong SAR, China

**Keywords:** oocyte, follicle development, cyclophosphamide, mitochondria, ClpP

## Abstract

Chemotherapy is extensively used to treat cancers and is often associated with ovarian damage and leads to premature ovarian insufficiency and infertility, while the role of mitochondria during ovarian damage with chemotherapy remains unknown. This study used a mouse model with oocyte-specific deletion of mitochondrial stress response gene Caseinolytic peptidase P (*Clpp*) to investigate mitochondrial homeostasis in oocytes from mice receiving a chemotherapeutic drug cyclophosphamide (CTX). We found that oocyte-specific deletion of *Clpp* reduced fecundity of the mice at advanced age. The deletion led to meiotic defects with elevated abnormal spindle rate and aneuploidy rate with impaired mitochondrial function in the MII oocytes from 8-week-old mice. Upon CTX treatment at 8-week-old, the oocyte competence and folliculogenesis from the oocyte-specific *Clpp* knockout mice was further deteriorated with dramatic impairment of mitochondrial distribution and function including elevated ROS level, decreased mitochondrial membrane potential, respiratory chain activity and ATP production. Taken together, the results indicate that that ClpP was required for oocyte competence during maturation and early folliculogenesis, and its deficiency deteriorate cyclophosphamide-induced ovarian damage.

## Introduction

There is a raising trend of cancer incidence at young age especially breast cancer worldwide in the past decade (1–3). Chemotherapy has significantly improved the survival of cancer patients but has also led to severe damage of the ovary in females. Female survivors at reproductive age have a high chance of developing premature ovarian insufficiency and losing their fecundity after chemotherapy. There are diverse types of chemotherapeutic agents that inhibit proliferation and induce death of malignant cells (4). Among those, the alkylating agent cyclophosphamide (CTX) has been widely applied to treat cancers especially breast cancer and leukemia, which have a high incidence in reproductive age women. Phosphoramide mustard is an active metabolite of CTX and exerts anti-tumor effects by inducing DNA crosslinks and inhibiting DNA replication and transcription (5). It also impairs mitochondrial membrane potential and induces accumulation of cytochrome C in the cytoplasm to interfere tumor cell proliferation and induce apoptosis (4).

Mitochondria are unique organelles in the cytoplasm of most eukaryotic cells, they are considered to be originated from specialized bacteria that survive endocytosis and become endosymbiotic in host cells during evolution (6). The major function of mitochondria is production of energy for cellular activities. Within the mitochondrial matrix, oxidative phosphorylation (OXPHOS) synthesizes ATP continuously to drive many biological processes in living cells (7). The mitochondria also play critical roles in other cellular activities, such as calcium signaling, cell cycling, differentiation, senescence and cell death (8–10). Moreover, mitochondria are reported a close relationship with endocrinology such that they were suggested to play essential roles in steroidogenesis and the synthesis of the stress hormones (11–13). A unique feature of mitochondria is that they contain their own DNA, namelymitochondrial DNA (mtDNA). The mtDNA is a double-stranded circular DNA with a length of 16.6 kb. It contains 37 genes that encode 13 proteins, 22 transport RNAs, and 2 ribosomal RNAs participating mainly in OXPHOS (14). Each mitochondrion contains 2-10 copies of mtDNA, and there are 100-10000 mitochondria in each somatic cell (15). Mitochondria have protective functions for cellular processes and dysfunctional mitochondria have been implicated in several human disorders and conditions, such as mitochondrial diseases (16), cardiac dysfunction (17), heart failure (18) and aging (19, 20). Most recently, mitochondria are also found to participate in mRNA storage for oocyte competence and final maturation (21).

Ovarian follicles are the basic functional units of ovary. Each follicle consists of a female germ cell and surrounding somatic cells. Precise cooperation of the two cell types is needed to maintain the reproductive and endocrinological functions of the ovaries (22). The number of mitochondria changes at different stages of oogenesis and ranges from 10 to 100,000. Loss of functional mitochondria in oocytes leads to follicle atresia, oocyte maturation disturbance and accelerated ovarian aging (23). While mitochondrial dysfunction in somatic sells is associated with follicle atresia, defects in oocyte maturation, fertilization and decreased blastocyst formation (23, 24).

Mitochondrial unfolded protein response (mtUPR) is a key component of the mitochondria quality control regulatory network, which is determinative for normal mitochondrial functions (25). Caseinolytic peptidase P (encoded by *Clpp* gene) plays an imperative role in mtUPR and it is activated when unfolded or misfolded proteins accumulated in the mitochondrial matrix (26, 27). The activated ClpP cleaves the misfolded or unfolded proteins into smaller peptides, which are then translocated into the cytoplasm to re-balance the mitochondria proteostasis (26, 28). *Clpp* mutations in human were reported cause the Perrault syndrome, an autosomal recessive disease associated with sensorineural hearing loss and premature ovarian failure (29). Mice with global germline deletion of *Clpp* exhibit growth retardation, hearing loss, decreased pre- and postnatal survival, and female infertility (30). Our previous study found that female mice lacking *Clpp* were infertile, produced fewer mature eggs and two-cell embryos and failed to produce blastocysts (27). The mutant mice ovaries showed accelerated follicular depletion, consistent with diminished ovarian reserve (27). However, it is still not clear whether these defects are due to a direct effect of mitochondrial dysfunctions of the germ cells or follicular cells in the ovary, or a secondary effect of mitochondrial dysfunctions in extra-ovarian tissues. Global *Clpp* deletion led to smaller mitochondria with low aspect ratio (length/width), decreased membrane potential and elevated ROS level in our previous study (27). Similarly, chemotherapy increases the ROS level and induces the mtDNA damage (31, 32). The functions of *Clpp* in ovary during chemotherapy damage are not known yet. In this study, we generated a conditional knockout mouse model to delete *Clpp* specifically in oocytes, we aim to investigate the role of *Clpp* in maintaining mitochondrial functions in follicle development and oocyte maturation. Moreover, we explored the effect of *Clpp* deletion on the aggravation of chemotherapy-mediated ovarian damage.

## Materials and methods

### Animal and genotyping

**Figure 1:**
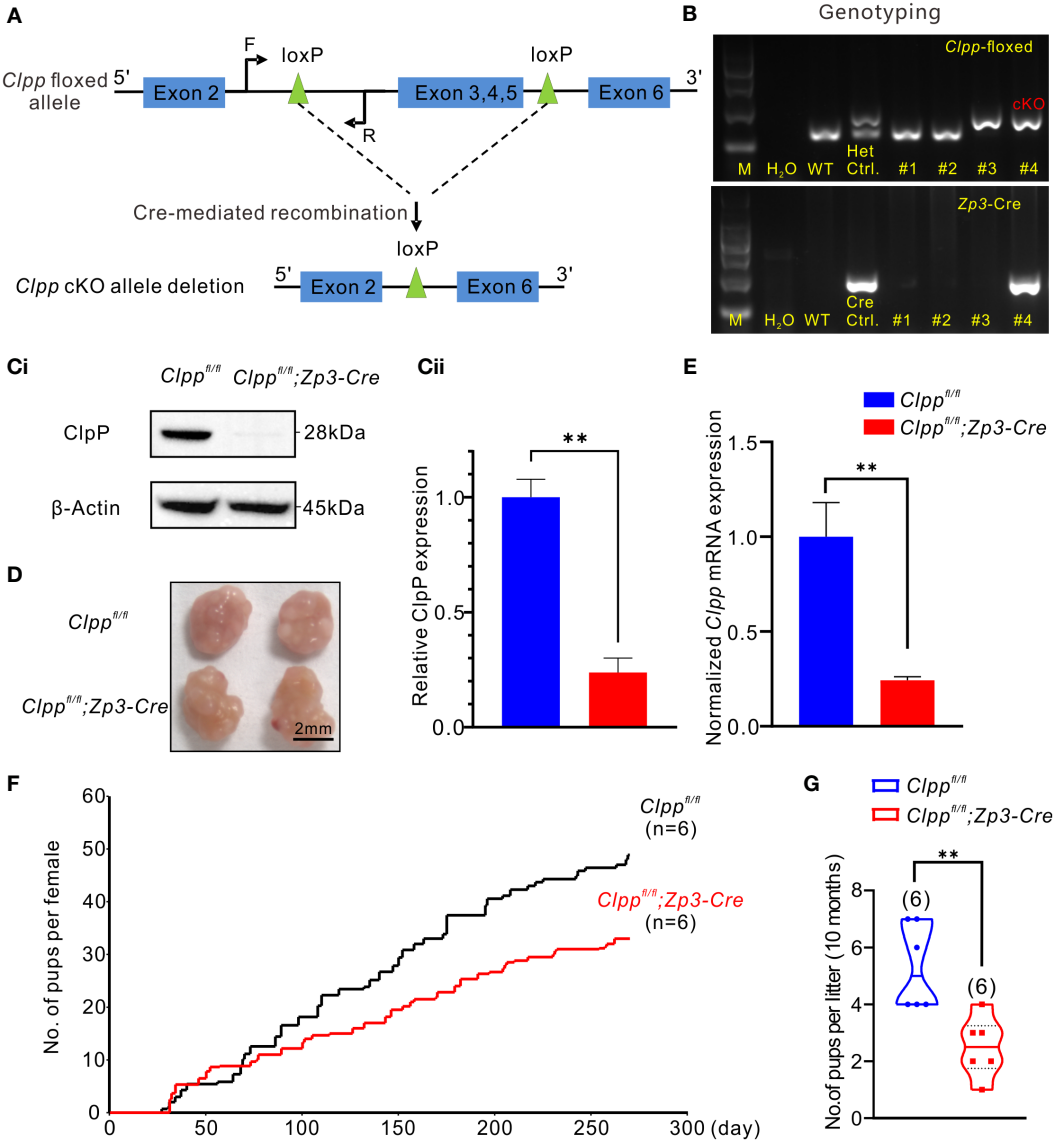
*Clpp* deletion reduced fertility efficiency along with aging. **(A)** Design of the Cre- LoxP cKO system in the *Clpp* allele, LoxP sites were inserted on both sides of exon 3/4/5, the two LoxP sites were recombined under the action of CRE protein, and exon 3/4/5 in the two LoxP sites were knocked out. (F=forward primer, R=reverse primer). **(B)** Genotyping of the *Clpp*; *Zp3*-Cre cKO mice. Wild-type (WT) mice tail DNA was used as negative control for PCR analysis. *Clpp^fl/+^* (Het.) mice tail DNA was used as a positive control, and a *Zp3*-Cre mice tail DNA was used as a positive control for Cre expression. DNA from different mice tail (e.g., #1-#4) was extracted and underwent PCR analysis to check the insertion of LoxP as well as the successful Cre expression. M means marker. **(Ci, Cii)** Western blotting analysis showed the ClpP protein level at 2-month-old mice in the GV oocytes. β-Actin was used as a loading control. **(D)** Image of representative ovaries isolated from control and cKO mice (8 weeks old, n=3), The scale bar is 2 mm. **(E)** RT-PCR analysis showed the *Clpp* mRNA expression level in GV oocytes. **(F)** Fertility tests showed the average on accumulated number of pups per female up to 10 months and **(G)** number of pups per litter in control and cKO groups at 10 months. All the oocyte and ovary samples were from 8-week-old female mice. **p < 0.01. The results represent mean ± SEM (standard error of the mean). Significance was determined by Student *t* test.

All the mice used were of the C57BL/6J background and were kept in the animal facilities at the Peking University Shenzhen Hospital. The *Clpp^fl/fl^* mice were produced by the CRISPR/Cas9 and homology-directed repair (HDR) techniques. Two LoxP sites were inserted into the mouse genome to target exon 3-exon 5 of *Clpp* for deletion (**Figure 1A**). Two guide RNAs (gRNA), donor vector containing the LoxP sites, and Cas9 mRNA were co-injected into fertilized mouse eggs to generate the targeted offspring. The F_0_ founder animals were identified by PCR and sequencing analysis. The mice were then bred with wild-type (WT) mice to generate the F_1_ generation and to test for germline transmission. The gRNA sequences used are listed in **Supplementary Table 1**. The *Zp3*-Cre mice were then crossed with the *Clpp^fl/fl^* mice to generate an oocyte-specific *Clpp* knockout mouse line. The mice were housed in a 12-hour light-dark cycle with free access to water and food. All experimental protocols were approved by the ethics committee of the University of Hong Kong-Shenzhen Hospital.

The procedures of genotyping are detailed in a previous report (33). A small tissue piece was excised at the tail tip of mice and was lysed with 200 μL of digestion buffer (50 mM KCl,10 mM pH 9.0 Tris-HCl, 0.1% Triton X-100 and 0.4 mg/ml proteinase K) at 55°C overnight, and then at 98°CC for 10 min to denature the proteinase K in the tail lysate. PCR analysis was performed using the Rapid Taq polymerase (Cat. P222-02, Vazyme, Nanjing, China) according to the manufacture’s protocol. The primers used for PCR are shown in **Table S1**.

The *in vivo* experimental design is as follow: the WT mice and cKO mice were divided into four groups: *Clpp^fl/fl,^ Clpp^fl/fl^* +CTX, *Clpp* ^fl/fl^; *Zp3*-*Cre* and *Clpp* ^fl/fl^; *Zp3*-*Cre* +CTX. We intraperitoneally injected 100 μL of 75 μM CTX once a time in *Clpp^fl/fl^* +CTX and *Clpp* ^fl/fl^; *Zp3*-*Cre*+CTX group. For the corresponding control group, we used the same injection method to administer 100 μL of PBS. All the experiments were conducted at least three independent biological replications. In this part, all the mice were given one-time injection at 8 weeks of age and collecting samples in 14 days later.

### Fertility test

For the fertility assessment, the *Clpp* cKO or WT female mice (age of mice: 8 weeks, n=6 for each group) were mated with WT male mice (age of mice: 8 weeks) in a mating ratio of 1♂:2♀, in 3 cages for each group. Fertility test will document female mice fertility between postnatal day 56 to postnatal day 280 (8 weeks to 40 weeks). The number of pups from each female was recorded for fertility analysis.

### Tissue collection and histological analysis

The *Clpp* cKO and WT female mice (8 weeks old, n=3 for each group) were euthanized by cervical dislocation. The ovaries were immediately collected and fixed in 4% (w/v) paraformaldehyde with phosphate-buffered saline (PBS, Cat. C10010500BT, Thermo Fisher Scientific, MA, USA) overnight before dehydration, paraffin embedding, sectioning at 5 μm thick and mounting on glass slides. The samples were then deparaffinized, stained with hematoxylin solution for 90 sec, washed three times with ddH_2_O, mounted with neutral balsam (Cat. G8590, Solarbio, Beijing, China) and imaged under a light microscope. Follicles at different developmental stages were counted in the whole ovaries to determine the total number. Primordial, primary, secondary, antral and atretic follicles were classified as described previously (34).

### Oocyte and embryo collection

Mouse oocytes and embryos were collected using standard protocol (27). Female mice were injected with 10 IU PMSG (Cat. P9970-1000 Solarbio, Beijing, China) intraperitoneally to stimulate follicular development. An injection of 10 IU of hCG (Cat.NB1122, NSHF, Ningbo, China) was given at 48 hours post-PMSG injection to induce oocyte maturation and ovulation. Follicles, cumulus-oocyte complex (COCs) or embryos were collected at different time points: (1) at 44 hours post-PMSG injection, ovaries were collected into M2 medium (Cat. M7167, Sigma, MO, USA), ovarian follicles were isolated with a disposable syringe, and GV oocytes were collected from the follicles after removal of the granulosa cells using a pipette with an internal diameter of 75 μm; (2) at 14-16 hours after hCG injection, COCs were collected from the oviduct, and MII oocytes(n=10/15) were isolated after removal of the cumulus cells by incubation in M2 medium containing 1 mg/mL hyaluronidase (Cat. H3506, Sigma, MO, USA) followed by aspiration through a pipette; (3) at 44-48 hours and 92 hours post-hCG injection, 2-cell embryos(n=3) and blastocysts(n=6/7), respectively were collected from the oviduct of mated female mice that were caged with 12-week-old WT male mice immediately after hCG injection. Mating was confirmed by the presence of vaginal plugs in the following morning.

### Oocyte immunofluorescence staining

Oocytes were fixed in 4% (w/v) paraformaldehyde in PBS for 30 min, and permeabilized in 0.5% Triton X-100 for 5 min before incubation with 2 μg/mL Alexa Fluor 488 conjugated-anti-α-tubulin antibody (Cat.16-232, Millipore, Billerica, MA, USA) for 1 h, washed three times for 5 min in PBS and stained with 4’,6-diamidino-2-phenylindole (Cat#G1012-10ML DAPI, Servicebio) prior to being examined under a Zeiss LSM 900 confocal microscope. The tubulin staining was examined with an excitation at 488 nm and an emission at 530 nm while that for DAPI with an excitation at 350 nm and an emission at 470 nm and female mice (8 weeks old, n=5 for each group) were used for oocyte sample collection.

### Evaluation of mitochondrial morphology

Oocytes were incubated in M2 medium containing 500 nM cell permeant MitoTracker™ Red CMXRos (Cat. M7512, Invitrogen, MA, USA) for 30 min at 37 °C in a dark environment with 5% CO_2_ in air. After washing three times with fresh M2 medium (2 min each time), the oocytes were mounted on non-fluorescent glass slides for imaging. The images were captured by a Zeiss LSM 900 confocal microscope.

### Quantification of mtDNA copy number in oocytes

Female mice (8 weeks old, n=3 for each group) were used for oocyte sample collection for this assay. To quantify mtDNA copy number in MII oocytes, the Cox3 fragment was amplified and subcloned into the pCR™2.1-TOPO^®^ - cloning vector (Invitrogen, MA, USA) as previously described (35). The primer sequences used are listed in **Supplementary Table S1**. The One Shot TOP10 competent cells were used for plasmid transformation. After overnight incubation at 37 °C, the recombinant plasmids were extracted using the Qiagen plasmid isolation kit (Cat.12145, Qiagen, Hilden, Germany). The inserted mtDNA fragment was confirmed by DNA sequence analysis. The plasmid DNA was quantified using a NanoDrop 2000 spectrophotometer (Thermo Scientific, MA, USA). A standard curve from 10^8^ to 10^1^ plasmid molecules was generated by serial 10-fold dilutions. Single MII oocyte was lysed in 10 μL of lysis solution before incubation at 55°C for 2 hours. The proteinase K in the lysis solution was denatured by heating at 95°C for 10 min and the mixture was used directly for downstream PCR in triplicates for each group. Each 10 μL reaction mixture contained 5 μL of Taq Pro Universal SYBR qPCR Master Mix (Cat. Q712-02, Vazyme, Nanjing, China), 0.3 μM primers, and oocyte DNA. The mtDNA copy number of each oocyte was then extrapolated from the standard curve.

### Determination of reactive oxygen species level

6-carboxy-2’, 7’-dichlorodihydrofluorescein diacetates (carboxy-H2DCFDA; Cat. C-400, Life Technologies, Thermo Fisher Scientific, MA, USA) was used to assess the ROS levels in mouse oocytes (36). The MII oocytes were pre-treated with 10 mM H_2_O_2_ in M2 medium for 5 min to induce ROS generation. They were then washed and incubated with 30 µM H2DCFDA in M2 medium for 20 min. The oocytes were washed 3 times with M2 medium and then immediately imaged under a Zeiss LSM 900 confocal microscope. The Image J software was used to quantify the fluorescence intensity.

### Evaluation of mitochondrial membrane potential

Mitochondrial membrane potential was evaluated with the JC-1 probe (Cat. T3168, Invitrogen, MA, USA). In brief, oocytes were incubated with M2 medium containing 2 μg/mL JC-1 probe for 30 min at 37°C in the dark and then washed 3 times in M2 medium for 3 mins each time. The oocytes were immediately examined under a Zeiss LSM 900 confocal microscope. The JC-1 dye exhibits a potential-dependent accumulation in mitochondria as indicated by an emission shift of fluorescence from green (~529 nm) to red (~590 nm). Mitochondrial depolarization was determined by the ratio of red-to-green fluorescence intensity.

### Quantitation of ATP

The ATP content of individual oocyte was determined using the ATP Bioluminescent Somatic Cell Assay Kit (Sigma, MO, USA). Each oocyte was mixed with 100 µL of somatic ATP release reagent and 100 μL of ATP mix working solution (diluted 1:25 from ATP Assay Mix stock solution) before loading onto the 96-well plate and incubation at room temperature for 3-5 min to allow hydrolysis of endogenous ATP. The amount of emitted light was immediately measured with a BioTek luminometer (Cat. SYNERGY H1, BioTek, Vermont, USA). Background luminescence was subtracted from all readings. ATP in individual oocytes was determined by comparison to a standard curve generated in the range z.

### RNA isolation and quantitative reverse transcription PC

Total RNA was extracted from 20 oocytes per mice using RNeasy^®^ Micro Kit (Cat. 74004, Qiagen, Hilden, Germany) and reverse transcription was performed using the HiScript III RT SuperMix for qPCR (+gDNA wiper) kit (Cat. R323-01, Vazyme, Nanjing, China) in two steps. First, template RNA and 4 × gDNA wiper mix were incubated at 42°C for 2 min to remove genomic DNA contamination. The 5X HiScript III qRT SuperMix was then added and the reverse transcription was carried out at 37°C for 15 min and end at 85°C for 5 sec. RT-qPCR was carried out on a Life 7500 (Cat. 4351107, Applied Biosystems™, Thermo Fisher Scientific, MA, USA). Complementary DNA (cDNA) was prepared as described above. Each experiment was repeated at least three times using different animals. The reaction mixture contained 10 µL of the Taq Pro Universal SYBR qPCR Master Mix (Cat. Q712-02, Vazyme, Nanjing, China), 7 µL of H_2_O, 1 µL of primers, and 2 µL of cDNA. The 2^-ΔΔCT^ (CT: cycle threshold) method was used to calculate relative expression levels after normalization to β-actin mRNA expression. The primers used are listed in **Table S1**.

### Chromosome spreading

Oocytes were incubated in Tyrode’s buffer (pH 2.5) for 30 s at 37°C to remove the zona pellucida. After washing 4 times in M2 medium, the oocytes were fixed in a drop of 1% paraformaldehyde with 0.25% Triton X-100 (Cat. 85111, Thermo Fisher Scientific, MA, USA) and 6 µM DTT (Cat. R0862, Thermo Fisher Scientific, MA, USA) on a glass slide. After air drying, chromosomes were counterstained with DAPI and examined under a confocal microscope.

### Protein extraction and western blotting analysis

The sample of GV oocytes were collected from WT and ClpP cKO female C57BL/6 mice and suspended in lysis buffer [50mM HEPES-KOH (pH 7.5), 100mM KCl, 2mM EDTA, 10% glycerol, 0.1% NP-40, 10mM NaF, 0.25mM Na3VO4, and 50mM ßglycerophosphate] supplemented with complete protease inhibitor (Cat. 04693116001, Roche, Basel, Switzerland). The samples were homogenized and centrifuged at 20,000 g for 20min at 4°C, after which the supernatant was retained for western blotting analysis. The proteins in each sample were separated using 8–16% Bis-Tris gels (Cat. M00659, SurePAGE™, GenScript, Nanjing, China) and a mini protein electrophoresis system (Cat. 1658034, BIO-RAD, CA, USA) following the manufacturer’s instructions. The protein bands were then transferred to polyvinylidene fluoride (PVDF) membranes (Cat. IPVH00010, Immobilon, Millipore, MA, USA) *via* a Mini Trans-Blot Electrophoretic Transfer Cell (Cat. 1703930, BIO-RAD, CA, USA). The immunoreactive bands were detected and analyzed with a Bio-Rad ChemiDoc MP imaging System (Cat. 12003154, BIO-RAD, CA, USA) in conjunction with the Image Lab Software (Bio-Rad, CA, USA). The relative protein levels in each sample were normalized to ß-Actin to standardize the loading variations. The obtained images were analyzed by image J software for gray value analysis.

### Statistical analysis

The results displayed by numerical values are combined with the results of three independent repeated experiments for *t*-test; for the data displayed by ratios, the ratio of each group is calculated separately after each independent repeated experiment, and then the *t*-test is performed to evaluate the statistic difference between two groups. Results showed in the figures were given as the mean ± SEM. At least three independent samples were repeated in all experiments. Groups were compared using the two-tailed unpaired Student’s *t*-tests. Statistically significant *P*-values (< 0.05, < 0.01 and < 0.001) were indicated by asterisks (*, ** and ***, respectively), while ‘ns’ represents not significantly different. Graphs were generated using the Microsoft Excel and GraphPad prism 8. Figures were prepared with the CorelDraw version X8 (Corel Corp., Ottawa, ON, Canada).

## Results

### Oocyte-specific knockout of ClpP reduced fertility efficiency along with aging

The *Clpp* ^fl/fl^; *Zp3*-Cre mice were identified by genotyping (**Figure 1B**). The 213-base pair (bp) band (higher band in **Figure 1B**) of the heterozygous (Het.) control indicated that the LoxP sites were inserted into one strand of the genomic DNA. The unedited strand of the genomic DNA produced a 150 bp band (lower band in **Figure 1B**) by PCR. The homozygous floxed *Clpp* mice exhibited only a single 213 bp band (**Figure 1B**). The mice were also genotyped for a Cre expression, which was indicated by a 293 bp band in the gel (**Figure 1B**). The homozygous floxed *Clpp* mice that expressed Cre (*Clpp* ^fl/fl^; *Zp3*-*Cre*) represented the ClpP conditional knockout (cKO) mice. The wild-type (WT) control mice used were with the genotype of *Clpp* ^fl/fl^ or *Clpp* ^fl/+^ and were from the same litter of the cKO mice.

We determined the mRNA and protein levels of *Clpp* in the oocytes of the cKO mice to confirm the knockout efficacy. The result of RT-qPCR demonstrated a decreased *Clpp* mRNA level in the *Clpp* cKO oocytes, and the *Clpp* mRNA level of the control was approximately 4 times that of the cKO mice (**Figure 1E**). Western blot analysis demonstrated a band for ClpP expression in the control oocytes, and no band was detected in the cKO oocytes (**Figures 1Ci, Cii**). The results confirmed successful knockout of ClpP in the oocytes of the *Clpp^fl/fl^; Zp3*-*Cre* mouse line. Next, we evaluated the fertility of the mice. At 8-week-old, the morphology and size of the ovary were comparable between the control and the *Clpp* cKO mice (**Figure 1D**). The *Clpp* cKO mice were fertile (**Figure 1F**) but the number of pups per litter in the *Clpp* cKO female mice decreased with aging (**Figure 1F**). At 10 months of age, the litter size was significantly lower in the cKO mice than the controls (**Figure 1G**).

**Figure 2:**
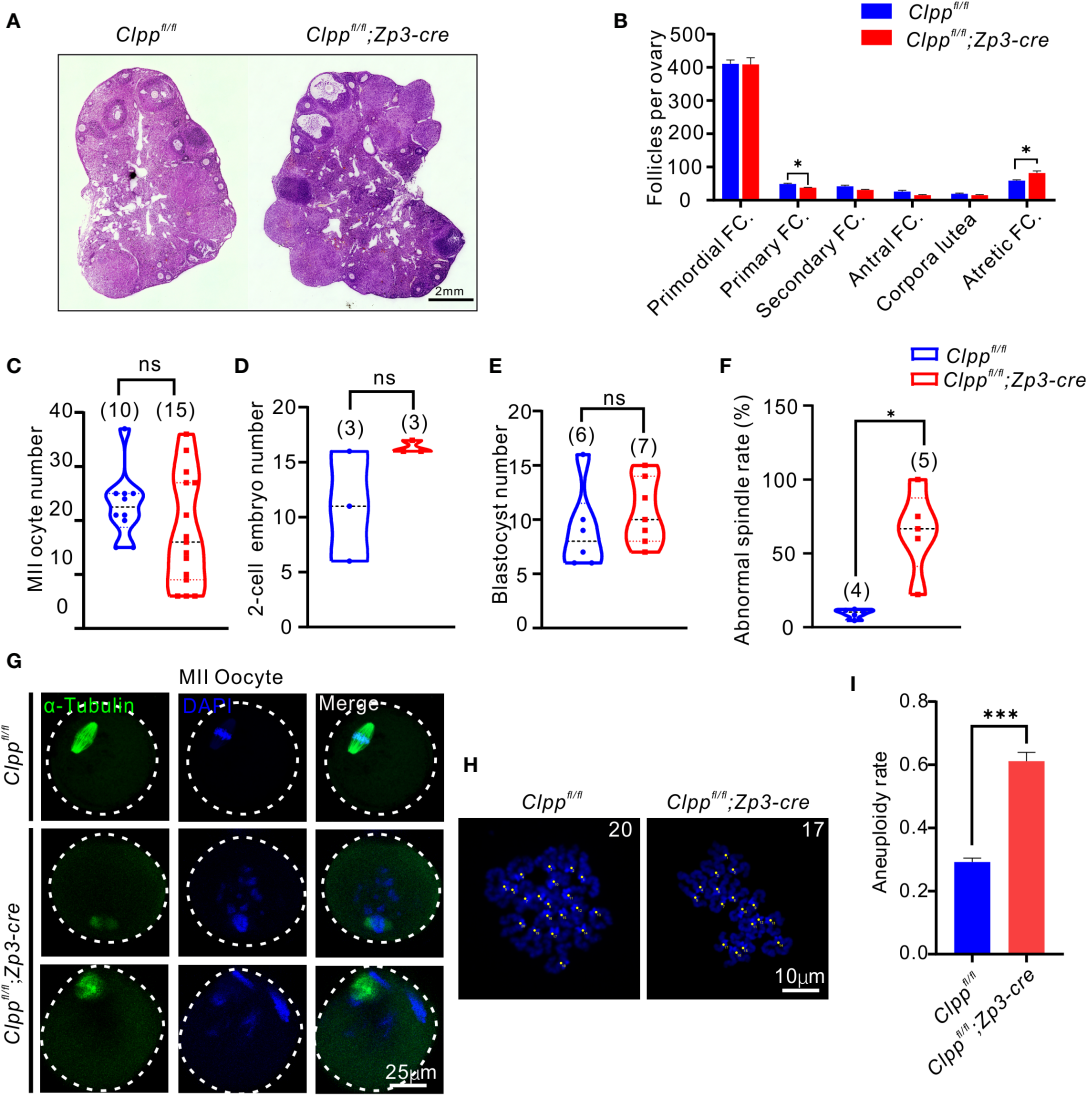
*Clpp* ^fl/fl^; *Zp3*-Cre cKO female mice exhibits oocyte meiotic defects. **(A)** Ovarian histomorphological analysis in 8-week-old *Clpp^fl/fl^*; *Zp3*-Cre cKO and control female mice. **(B)** Follicle counts were performed using three biological replicates for each group(n=3). **(C)** Mature (MII) oocyte, **(D)** two-cell embryo, and **(E)** blastocyst generation in 8-week-old control and *Clpp^fl/fl^*; *Zp3*-Cre cKO female mice. **(F)** Representative spindles from *Clpp^fl/fl^*; *Zp3*-Cre and control MII oocyte obtained *in vivo*. Column 1, anti-a-tubulin antibody (green); Column 2, DAPI (blue); Column 3, merged images of DAPI and anti-a-tubulin. **(G)** Percentage of *Clpp^fl/fl^*; *Zp3*-Cre and control MII oocyte with abnormal chromosome alignment on spindle. **(H)** Chromosome staining of *Clpp^fl/fl^*; *Zp3*-Cre and control MII oocytes with DAPI and **(I)** ratio of chromosomal aneuploidy. All the oocyte and ovary samples were from 8-week-old female mice. N.S. means no significant difference; *p < 0.05; ***p < 0.001. The results represent mean ± SEM. Significance was determined by Student *t* test.

We further studied the ovarian histology of the 8-week-old *Clpp* cKO mice. The overall morphology of the cKO ovary was quite comparable with that of the control (**Figure 2A**). When the number of follicles at different stage of folliculogenesis in the whole ovary tissue was counted, we found a significant decrease in the number of primary follicles and a significant increase of the atretic follicles relative to that of the controls (**Figure 2B**). There was no difference in the number of follicles at other stages of folliculogenesis and corpora lutea between the two groups. In addition, the control and the *Clpp* cKO mice at 8-week-old produced similar number of MII oocytes, 2-cell embryos and blastocysts (**Figures 2C–E**).

### *Clpp* deletion affects meiotic progression in oocytes

We next evaluated the impact of ClpP deficiency on meiosis. The spindle of MII oocytes was visualized by α-tubulin immunostaining. The control oocytes showed well aligned spindle (green) and chromosomes (blue with DAPI), while the *Clpp* cKO oocytes exhibited a disrupted spindle formation and misalignment of spindle-chromosomes structure (**Figure 2F**). Quantitatively, the rate of abnormal spindles in the cKO oocytes was 64.78%, which was significantly higher than that of the control oocytes (9.36%, **Figure 2G**). We counted the number of chromosomes in these MII oocytes using the chromosome spreading assay (**Figure 2H**). Significantly, more aneuploid oocytes were found in the *Clpp* ^fl/fl^; *Zp3*-Cre mice than the *Clpp* ^fl/fl^ mice (**Figure 2I**; *p*=0.0021).

### Deletion of *Clpp* severely affects mitochondrial function in oocytes

**Figure 3:**
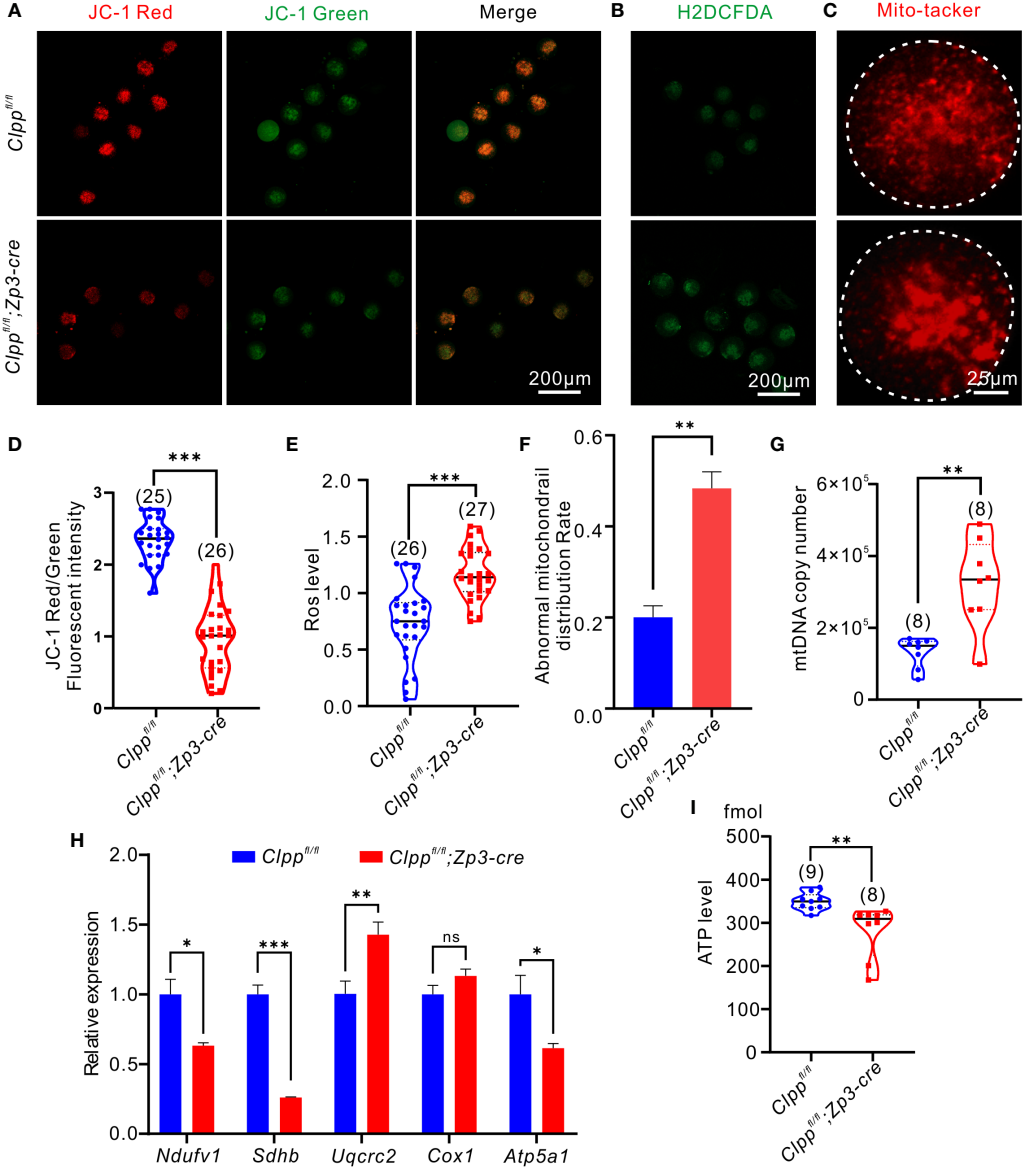
Oocyte-specific knockout of *Clpp* impairs mitochondrial function in MII oocytes. **(A)** Fluorescent micrographs of MII oocytes (8 weeks old, n=3) stained by mitochondria-specific probe JC-1. Red fluorescence represents aggregated Mitochondria and green fluorescence represents monomeric Mitochondria. **(B)** Using general oxidative stress indicator (Carboxy-H2DCFDA) to ROS level in *Clpp^fl/fl^*; *Zp3*-Cre and control MII oocyte (8 weeks old, n=3)following H_2_O_2_ treatment. **(C)** Mito-tracker was used to detect the distribution of mitochondria in oocytes (8 weeks old, n=3). **(D)** Mitochondrial membrane potential indicated by the red/green fluorescence intensity ratio. **(E)** The fluorescence pixel intensity of ROS levels in *Clpp^fl/fl^*; *Zp3*-Cre cKO and control MII oocyte following H_2_O_2_ treatment. **(F)** Ratio of abnormal morphological mitochondrial distribution in oocytes. **(G)** mtDNA copy number was determined by qPCR in MII oocytes collected from in *Clpp* ^fl/fl^ and *Clpp^fl/fl^*; *Zp3*-Cre mice. **(H)** Expression of respiratory chain genes was assessed using RT–PCR in GV oocytes collected from WT and *Clpp^fl/fl^*; *Zp3*-Cre cKO female mice. **(I)** ATP level in *Clpp^fl/fl^* and *Clpp^fl/fl^*; *Zp3*-Cre MII oocytes. N.S. means no significant difference; *p < 0.05; **p < 0.01; ***p < 0.001. All the oocyte and ovary samples were from 8-week-old female mice. The results represent mean ± SEM. Significance was determined by Student *t* test. *Atp5a1*, ATP synthase, H+ transporting, mitochondrial F1 complex; alpha subunit 1; *Cox1*, cytochrome c oxidase subunit I; *Ndufv1*, NADH dehydrogenase (ubiquinone) flavoprotein 1; *Sdhb*, succinate dehydrogenase complex iron sulfur subunit B; *Uqcrc2*, ubiquinol cytochrome c reductase core protein 2.

We hypothesized that *Clpp* deletion induced mitochondrial dysfunction and thereby causing spindle formation defect and high aneuploidy in oocytes. Therefore, we assessed the mitochondrial membrane potential, the driving force for mitochondrial ATP synthesis, by JC-1 staining. Mitochondria with high membrane potential exhibited red fluorescence, whereas those with low membrane potential exhibited green fluorescence (**Figure 3A**). The ratio of red to green signals in the *Clpp* ^fl/fl^; *Zp3*-Cre oocytes was significantly lower than that of the *Clpp* ^fl/fl^ oocytes (**Figure 3D**). We also evaluated the recovery ability from ROS stress in the *Clpp* cKO oocytes. The MII oocytes were pre-treated with H_2_O_2_ for 5 minutes before measurement of their ROS level by carboxy-H2DCFDA (**Figure 3B**). Compared with the *Clpp* ^fl/f^ oocytes, the ROS level in the *Clpp* ^fl/fl^; *Zp3*-Cre oocytes was significant increased (p<0.001, **Figure 3E**). We visualized the distribution of mitochondria using the Mito-tracker staining (**Figure 3C**), and found that the mitochondria in the *Clpp* cKO oocytes exhibited a strong aggregated pattern rather than an evenly distributed pattern as in the control oocytes (**Figure 3C**). The pattern was significantly higher in the cKO oocytes than the control oocytes (**Figure 3F**). Interestingly, the ClpP deficient oocytes had more mitochondrial DNA (mtDNA) copies than the control oocytes (**Figure 3G**). Furthermore, we measured the mRNA expression of five genes (*Atp5a1, Ndufv1, Cox1, Sdhb* and *Uqcrc2*) representing complex I-V in the mitochondrial respiratory chain by RT-qPCR. The results revealed a significant decrease of the *Atp5a1, Ndufv1, Sdhb* mRNA expression in the cKO oocytes when compared with the controls (**Figure 3H**). Not surprisingly, the ATP synthesis level was significantly decreased in the cKO oocytes (**Figure 3I**). These results demonstrated a disrupted oxidative phosphorylation in the ClpP cKO oocytes.

### Cyclophosphamide treatment deteriorated folliculogenesis and oocyte competence of *Clpp* ^fl/fl^; *Zp3*-Cre mice

**Figure 4:**
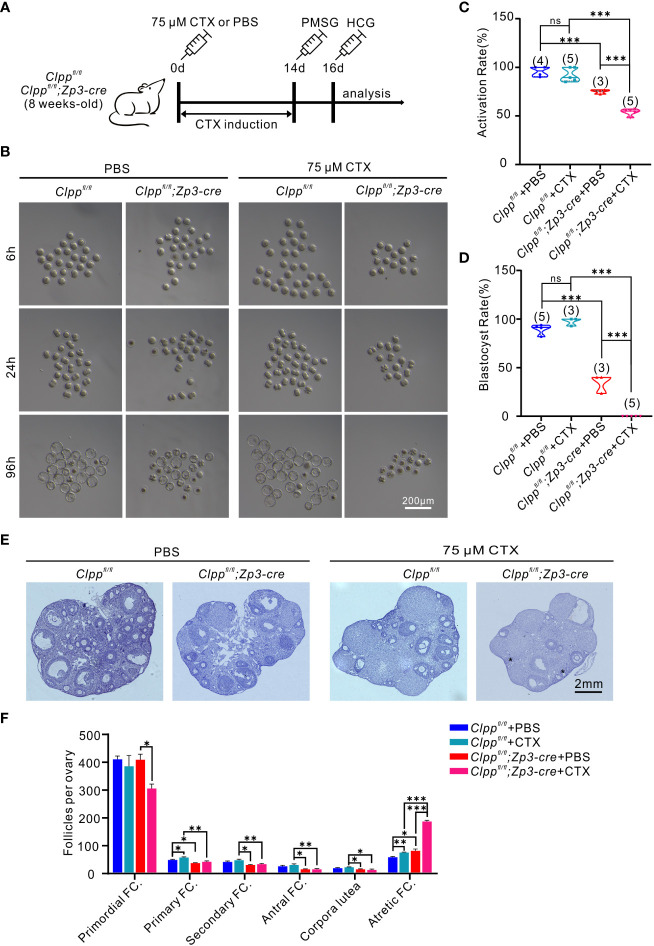
CTX deteriorated oocyte competence and follicle development in *Clpp^fl/fl^*; *Zp3*-Cre mice. **(A)** Schematic diagram showing the experimental design of cyclophosphamide injection. 8-week-old mice were injected intraperitoneally with 75 μM cyclophosphamide (CTX) at day 0. After 14 days of feeding on a normal diet, PMSG was injected on day 14 to promote follicle development, and HCG was injected on day 16 to excrete oocytes. **(B)** Representative images of different stages of MII oocytes after parthenogenetic activation obtained by stereomicroscope. **(C)** 6h After parthenogenetic activation, 2 pre-nucleolus observed was considered to be successfully activated. **(D)** The blastocyst rate showed the ratio of the total number of blastocysts and activated oocytes 96 hours after parthenogenetic activation. **(E)** Histomorphological analysis of mice ovaries after 14 days of cyclophosphamide treatment. **(F)** Folliculogenesis was compared among four groups. (n=3) All the oocyte and ovary samples were given one injection at 8 weeks of age. Continue feeding for 14 days after injection. N.S. means no significant difference; *p < 0.05; **p < 0.01; ***p < 0.001. The results represent mean ± SEM. Significance was determined by Student *t* test.

High-dose of CTX treatment causes premature ovarian insufficiency (POI) in mice ovaries (37). Here, we planned to use a reduced concentration of CTX to induce a mild stress to the ovary and test the role of *Clpp* in protecting the oocytes upon encountering environmental stress. We pre-tested the dosage of CTX and found 75 µM CTX induction will not affect the follicle development in the control *Clpp* ^fl/fl^ mice (**Figures 4E**, **F**). Thus, we decided to use this dosage and injected 75 µM CTX into the *Clpp* ^fl/fl^ and the *Clpp* ^fl/fl^; *Zp3*-Cre mice (**Figure 4A**). The number of follicles in the ovary of the *Clpp* ^fl/fl^; *Zp3*-Cre mice was obviously decreased in histological sections when compared with the PBS treated *Clpp* ^fl/fl^; *Zp3*-Cre mice or the CTX treated *Clpp* ^fl/fl^ mice (**Figure 4E**). Counting of the follicles in the whole ovary showed that the CTX treatment severely affected follicle development in the ClpP cKO mice; the numbers of primary, secondary and antral follicles were significant decreased, and the number of atretic follicles increased dramatically when compared with that of other groups (**Figure 4F**). The CTX treatment did not affect the development of the follicles of the *Clpp* ^fl/fl^ mice (**Figure 4F**).

Next, we studied the development competence of the treated oocytes. MII oocytes were collected from the treated mice 14 days later for parthenogenetic activation. The *Clpp* ^fl/fl^; *Zp3*-Cre+CTX oocytes failed to form blastocyst, whereas ~97% of the control oocytes from *Clpp* ^fl/fl^+CTX mice were able to form blastocytes (**Figures 4B, D**). Data analysis of three biological replicates showed that the CTX treatment significantly reduced the activation rate (**Figure 4C**) and blastulation rate (**Figure 4D**) of the *Clpp* ^fl/fl^; *Zp3*-Cre oocytes in the parthenogenetic activation assay.

### CTX aggravates the mitochondrial dysfunctions in the *Clpp* deleted oocytes

**Figure 5:**
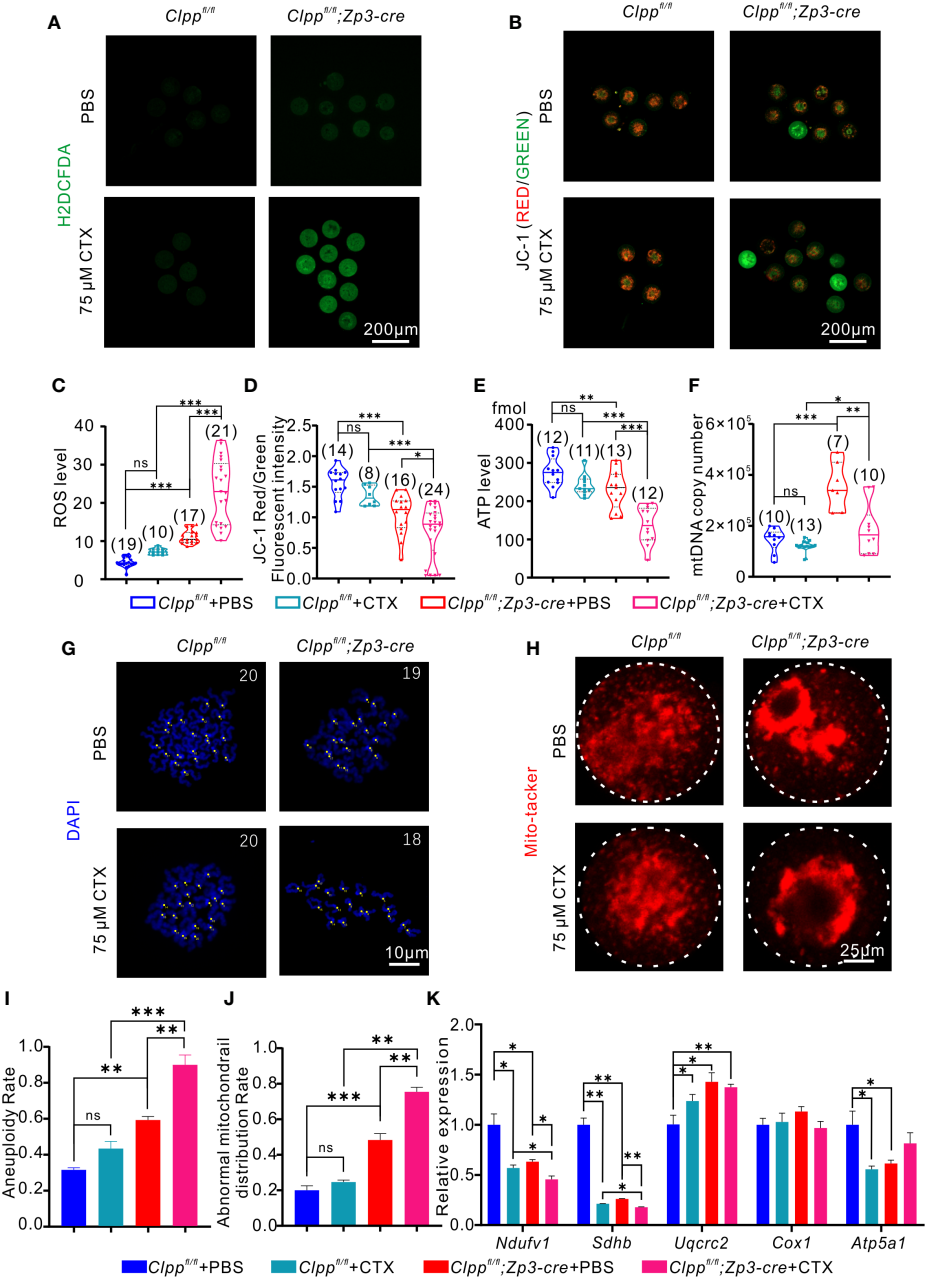
CTX induces oocyte deficiency by dramatically impairing mitochondrial function. **(A)** General oxidative stress indicator (Carboxy-H2DCFDA) was used to detect the ROS level in MII oocyte from each group following H_2_O_2_ treatment. **(B)** Fluorescent micrographs of MII oocytes stained by mitochondria-specific probe JC-1. Red fluorescence represents aggregated Mitochondria and green fluorescence represents monomeric Mitochondria. **(C)** The fluorescence intensity of ROS levels in MII oocyte from each group following H2O2 treatment. **(D)** Mitochondrial membrane potential (MMP) indicated by the red/green fluorescence intensity ratio. **(E)** ATP level and **(F)** mtDNA copy number was determined in MII oocytes collected from in *Clpp^fl/fl^/Clpp^fl/fl^* +CTX/*Clpp^fl/fl^*; *Zp3*-Cre and *Clpp^fl/fl^*; *Zp3*-Cre+CTX mice MII oocytes. **(G)** Chromosome staining of MII oocytes with DAPI. **(H)** Mito-tracker staining was applied to exhibit the distribution of oocyte mitochondria. **(I)** Ratio of chromosomal aneuploidy in oocytes from four groups. **(J)** Statistical analysis of abnormal mitochondrial distribution in oocytes from four groups after Mito-tracker staining. **(K)** Expression of respiratory chain genes were assessed using RT–PCR in GV oocytes collected from four groups of female mice. All the oocyte and ovary samples were given one injection at 8 weeks of age. Continue feeding for 14 days after injection. N.S. means no significant difference; *p < 0.05; **p < 0.01; ***p < 0.001. The results represent mean ± SEM. Significance was determined by Student *t* test.

We evaluated whether the CTX-induced phenotypes were due to a further deterioration of mitochondrial functions in terms of ROS level, mitochondrial membrane potential, ATP level and mtDNA copy number. The results showed a remarkably increase in ROS level (**Figures 5A, C**) and a decrease in red/green fluorescence intensity ratio (**Figures 5B, D**) in the *Clpp* cKO oocytes upon treatment with 75 µM CTX when compared with the PBS treated *Clpp* cKO oocytes. The treatment also significantly decreased the ATP level (**Figure 5E**) and the mtDNA copy number in the *Clpp* cKO oocytes (**Figure 5F**), indicating that the CTX treatment not only impaired mitochondrial functions but might also decreased the number of mitochondria in the Clpp cKO oocytes.

Although both the PBS and CTX treated *Clpp* cKO oocytes showed aggregation of mitochondria, the CTX treatment aggravated the abnormal distribution (**Figure 5H**) and the percentage of oocytes with the abnormal mitochondrial distribution was increased significantly in the CTX treated *Clpp* cKO oocytes relative to the PBS treated control oocytes (**Figure 5J**). The CTX treated *Clpp* cKO oocytes also exhibited significantly lower mRNA expression of *Ndufv1* and *Sdhb* when compared with the PBS treated counterparts (**Figure 5K**), suggesting that the treatment further affected mitochondrial oxidative phosphorylation in the *Clpp* cKO oocytes. As expected with a deterioration of mitochondrial function, the CTX treatment significantly increased the aneuploidy rate of the *Clpp* deficient oocytes but not the *Clpp* ^fl/fl^ oocytes (**Figures 5G, I**).

## Discussion

The previous studies have suggested *Clpp* plays a central role in mitochondrial unfolded protein response and is crucial to maintain protein homeostasis in the mitochondria (38–40). However, the precise role of *Clpp* in germ cell development is not fully understood. To our known, this is the first study investigated the impact of oocyte specific deletion of *Clpp* on folliculogenesis, oocyte competence and early embryo development. We found the specific deletion of *Clpp* in oocytes reduced fecundity of the mice at advanced age. The *Clpp* deletion led to meiotic defects and impaired mitochondrial distribution and function in the MII oocytes. CTX induction further affected oocyte competence in *Clpp* cKO mice and lead to an exhaustive disruption of blastocytes formation due to the severe effects on mitochondrial distributions and functions.

In the oocyte-specific *Clpp* cKO mice at 8-week-old, the fertility of the cKO mice was not different from the WT mice; they produced comparable numbers of GV oocytes, MII oocytes, 2-cell embryos, blastocysts and pups. However, the oocytes from *Clpp* cKO mice had increased aneuploidy rate and exhibited sign of reduced mitochondrial quality including decreased levels of mitochondrial membrane potential, mtDNA copy number and ATP synthesis, increased ROS level and mitochondrial aggregation, and changes in gene expression of the oxidative respiratory chain. These observations suggest that the phenotypic changes of mitochondria were not strong enough to affect the fertility significantly. The hypothesis is supported by the more severe phenotypes in the female mice of comparable age with global knockout of *Clpp*; they are infertile with reduced number of mature oocytes, moreover, the two-cell embryos are failed to form blastocysts (27). The severe phenotypes in the *Clpp* global knockout might be caused by a combined effect of *Clpp* deficiency in both the somatic cells and the germ cells. Indeed, the *Clpp*-deficient cumulus cells exhibit increased apoptosis and may negatively affect their cross talk with the oocytes in the COC leading to compromised oocyte competence (40). Our observations of a declined liter size of the cKO mice and an increased aneuploidy rate of oocytes with advanced age of the mice show that aging is another factor that enhances the detrimental impact of oocyte-specific knockout of *Clpp*. The observations are consistent with other studies reporting association of ovarian aging with high aneuploidy rate in human and mouse embryos and with reduced number of offspring (41, 42). It is noted that the *Clpp* global knockout mice exhibit an accelerated aging process with the increased loss of early-stage follicles (27). Further studies should be conducted to understand the aging factors that work with the *Clpp* deficiency in affecting oocyte competence.

We found that the *Clpp* cKO oocytes exhibited decreased mitochondrial membrane potential and ATP production, lower expression of genes coding for electronic transport chain (ETC) proteins, abnormal mitochondrial distribution and higher mtDNA copy number. Interestingly, the aneuploid human embryos also contained higher mtDNA copy number than the euploidy counterpart (43). Overall, both cellular energy metabolism and mitochondrial functions were severely affected in the *Clpp* deficient oocytes, which could be the reasons lead to elevated aneuploidy and decreased fecundity in the cKO mice.

CTX is an alkylating agent used as a cell cycle non-specific inhibitor for most treatment of breast cancers and leukemia. The hepatic cytochrome 450 enzyme converts CTX into two bioactive metabolites, 4-hydoxycyclophasphamide and acrolein (44, 45). Acrolein can directly induce mitochondrial oxidative stress by increasing the ROS level (32). CTX induces ROS in cells. It increases the level of H_2_O_2_ in the cerebrum and cerebellum (31). Mallard et al. found that chemotherapy with CTX reduced mitochondrial biogenesis, altered mitochondrial dynamics and potentiate mitophagy defects, exacerbated H_2_O_2_ production in skeletal muscles of breast cancer patients (21). In our study, we applied a relatively low dose of CTX (75 μM) to induce a gentle stress on the oocytes. The dose used induced a tolerable stress in the control oocytes but strikingly reduced oocyte competence and mitochondrial function in the *Clpp* deficient oocytes. These findings suggest the deletion of *Clpp* accelerate the CTX-induced stress to oocytes, decrease the tolerance level of mitochondria in *Clpp* cKO oocytes. The deficiency of *Clpp* in oocyte weakens the mitochondrial quality control regulation and CTX further broken the fragile balance of mitochondrial homeostasis and lead to the decline of oocyte quality.

We found an elevated aneuploidy rate in the *Clpp* deleted oocytes and the rate further increased after CTX treatment. Meiotic chromosome segregation error is previously reported as the major cause of aneuploid oocytes during ovarian aging (46). Mitochondria provide energy to support cellular events including spindle assembly, chromosome alignment and separation during oocyte meiosis (47). Mitochondrial dysfunction has been reported to be related to increase of aneuploidy in oocytes and embryos (48). ClpP is critical to maintain mitochondrial proteostasis (27). After *Clpp* deletion, the mitochondrial membrane potential dropped significantly and the ATP production was impaired in the oocytes, consistent with previous studies (30). CTX has been reported to impair oocyte quality leading to aberrant meiosis progression, abnormal spindle, and aneuploidy (49, 50). We had similar findings in the present study, in addition, we found CTX application led to more severe oocyte damage with significantly increased aneuploidy ratio and disrupted oocyte competence.

Histomorphometric assessment of the ovaries from the *Clpp* cKO mice and the control mice revealed reduced number of primary follicles and primordial follicles but a 2-fold increase of atretic follicles after CTX treatment in the cKO mice. These findings indicated that ClpP deletion induced mitochondrial stress and aggravated the follicle atresia from early stage with CTX treatment. However, this phenotype of early follicle loss in the *Clpp* cKO mice with CTX induction was not as strong as that during oocyte maturation. it indicated mitochondria might have more relevant protective role during oocyte maturation than folliculogenesis. These findings are consistent with current theory that mitochondria activity in oocytes from antral follicles was much higher than that of the pre-antral stage (51).

In this study, we used 8-week-old mice to investigate female fertility function in young mice, with the assessment lasting until an advanced age of 40 weeks. We then further investigated the damage to the ovaries caused by CTX treatment by selecting the group of 8-week-old mice, and evaluated folliculogenesis and oocyte competence two weeks after treatment, until the mice reached 10 weeks old. This design is consistent with our previous studies (27). We selected 8-week-old mice as the young group, as this is widely accepted as the age at which female mice are sexually mature and can produce healthy offspring (52, 53). We waited two weeks after CTX treatment to assess the damage, as we were interested in both short-term and long-term effects. There are known differences in ovarian function, hypothalamic-pituitary-ovarian axis, and reproductive outcomes between 8- and 10-week-old mice, and our methodology was designed with a paired age control group specifically to evaluate the damage caused by CTX on the ovaries of young mice. This design maximally eliminates potential systematic differences.

This study has several limitations. First, we did not evaluate the effect of CTX on ClpP deficient female mice at an advanced age. We observed a significant decline in the fecundity of ClpP deficient mice as they aged, producing fewer offspring. It would be interesting to investigate whether CTX aggravates ovarian damage during the aging process. Second, our current study indicates the important function of mitochondria during CTX damage. However, it does not provide direct evidence of the protective role of mitochondria in preventing ovarian damage from chemotherapy. Further studies could investigate the protective role by enhancing mitochondrial function or up-regulating mitochondrial functional genes in ovaries during CTX treatment. Lastly, while our study included some mechanistic approaches such as mitochondrial biogenesis, oxidative stress, mtUPR, and the role of other genes that regulate mitochondrial function, a more in-depth mechanistic approach is needed to provide a better understanding of the potential molecular mechanisms involved in the lack of mitochondrial regulating gene Clpp with damage to ovaries by CTX. It would be relevant to uncover the role of ClpP in maintaining follicle development and oocyte maturation, and its role in protecting the ovary from chemotherapy-induced damage is still challenging, especially in terms of providing mechanistic evidence.

In conclusion, *Clpp* is required for maintaining the oocyte competence during maturation and early folliculogenesis, targeted deletion of *Clpp* elevates aneuploidy rate, impairs mitochondrial function in oocytes and reduces number of primary follicles. Its deficiency deteriorate cyclophosphamide-induced ovarian damage.

## Data availability statement

The raw data supporting the conclusions of this article will be made available by the authors, without undue reservation.

## Ethics statement

The animal study was reviewed and approved by Ethical Committee of the University of Hong Kong-Shenzhen Hospital.

## Author contributions

Study design: TW, GL. Mice husbandry: JG, ZH, YX, RH, CG, JS, XZ. Mice experiments: JG, ZH, YX, TWu. Cellular and molecular experiments: GL, JG, YX, RH, CG, ZH, TWu. Figure compilation: JG, GL, TW, XL, CG. Funding acquisition: KL, WY, TW. Original manuscript draft: JG, GL, CG, TW. Manuscript review & editing: KL, WY, TW, GL, JG, CG. All authors contributed to the article and approved the submitted version.
