# Supplementary material for: Mitochondrial stress response gene Clpp deficiency impairs oocyte competence and deteriorate cyclophosphamide-induced ovarian damage in young mice
Source: Front Endocrinol (Lausanne). 2023 Mar 24;14:1122012. doi: 10.3389/fendo.2023.1122012 (PMC10081448; doi:10.3389/fendo.2023.1122012)

Fig 1-B agarose gel image

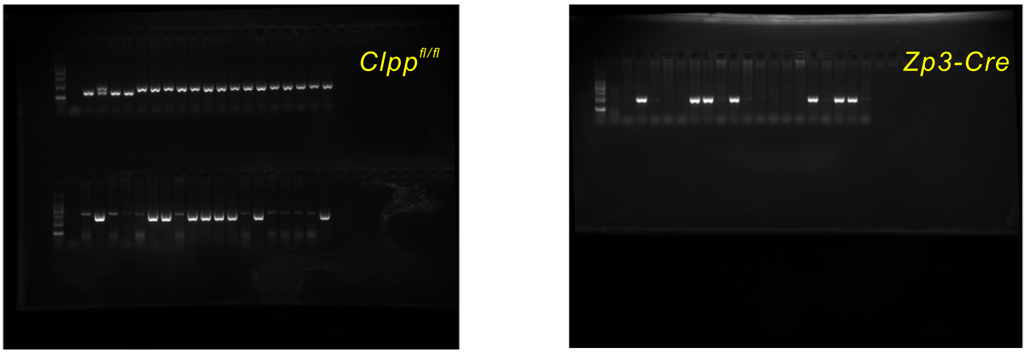

Fig 1-C Westren blot image

CLPP

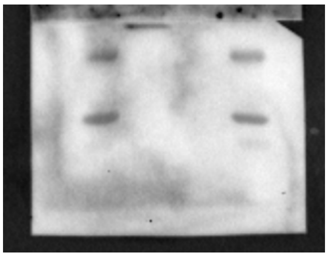

β-ACTIN

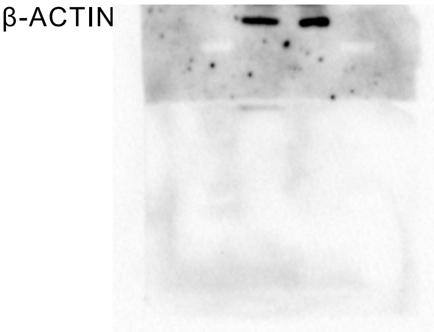

Fig 1-D Westren blot image

Clpp$^{fl/fl}$

Clpp$^{fl/fl}$;*Zp3-Cre*

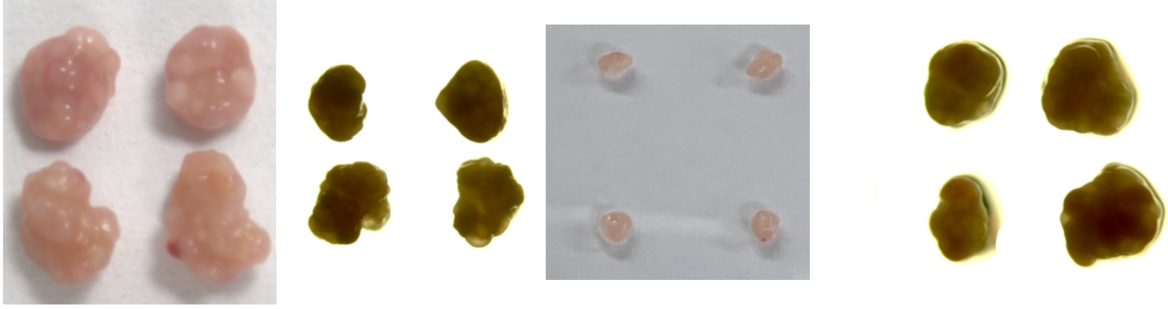

Fig 2-A Histological observation

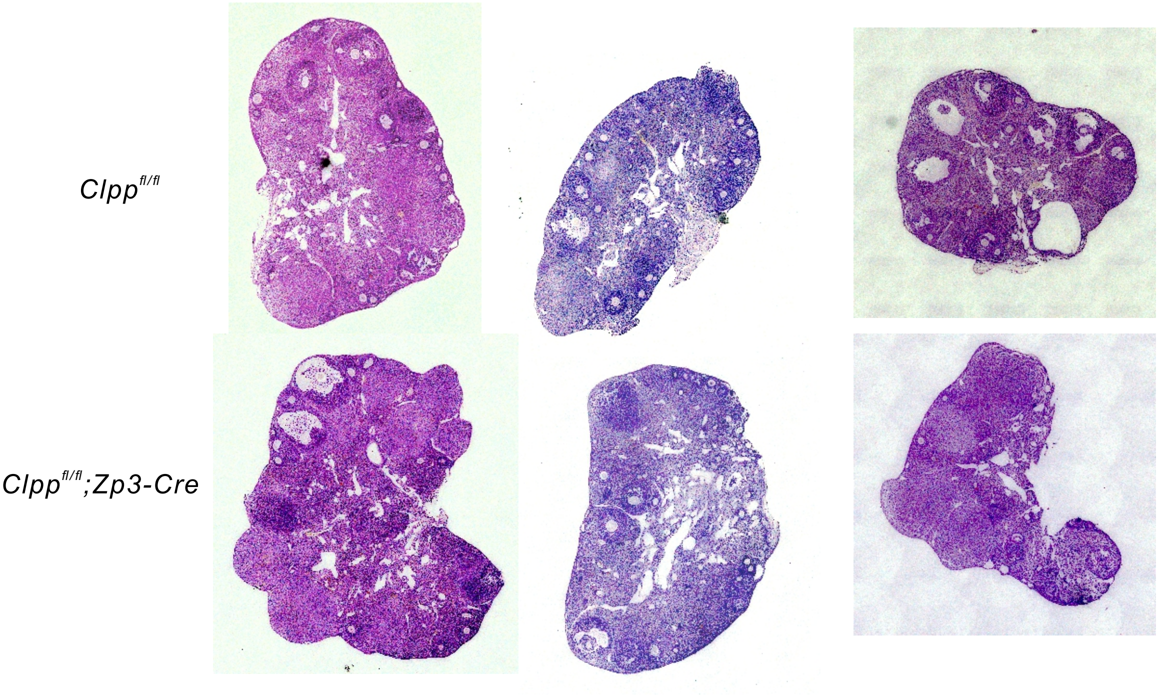

Fig 2-G Spindle image

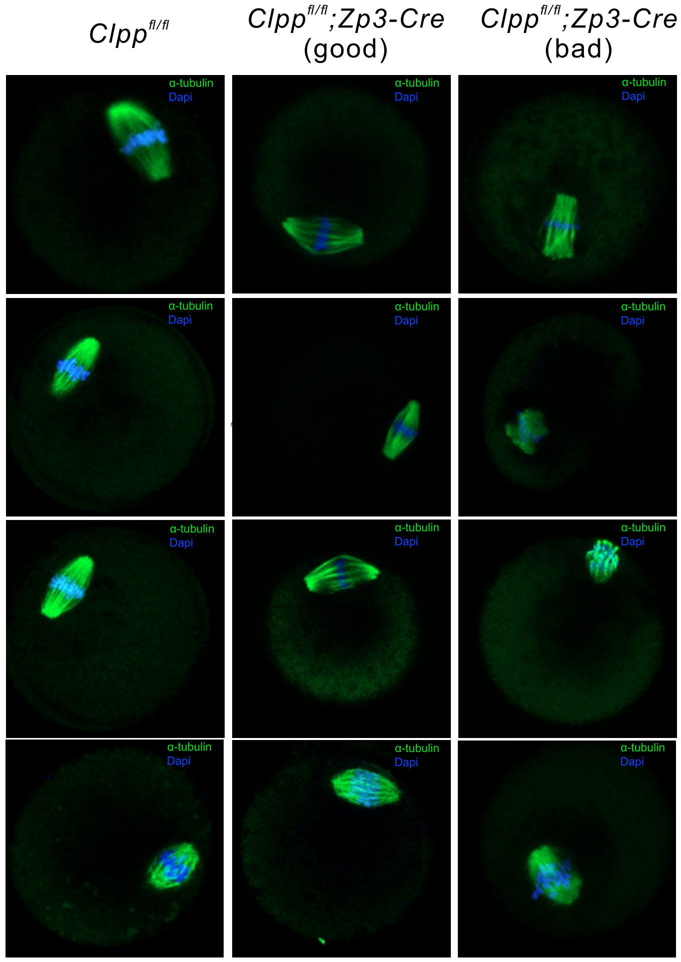

Fig 2-H Chromosomal spread image

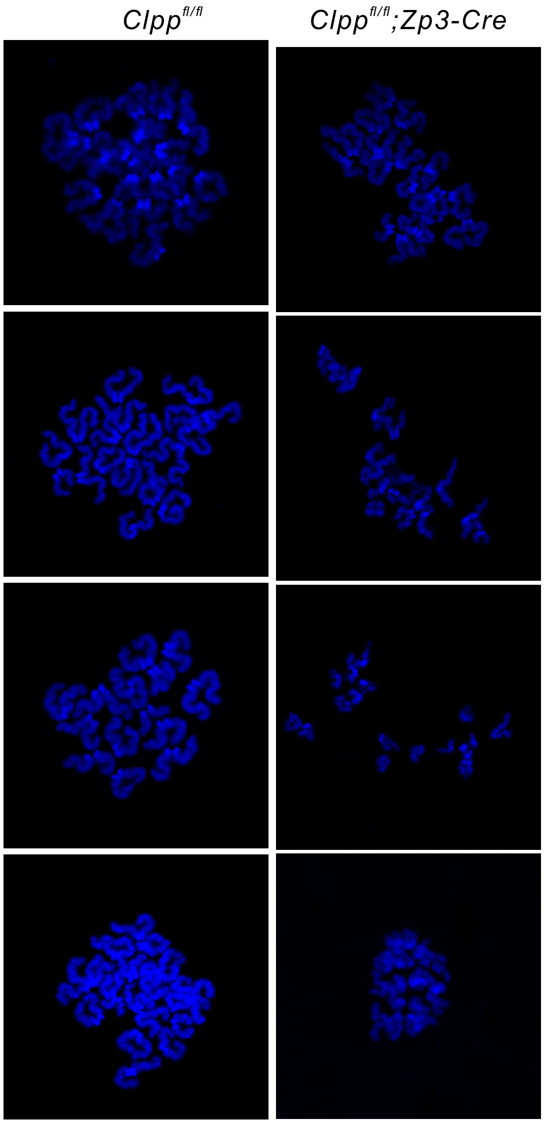

Fig 3-A Jc-1 staning

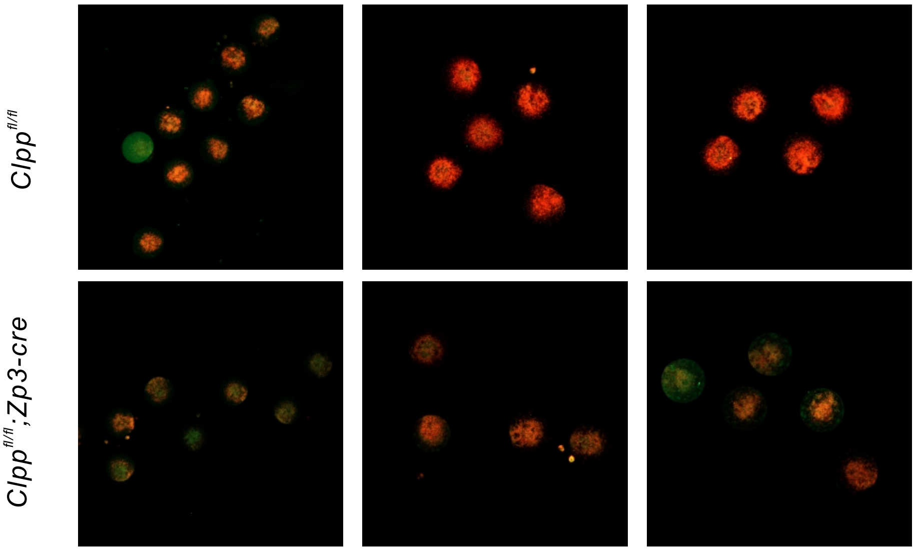

Fig 3-B ROS level staning

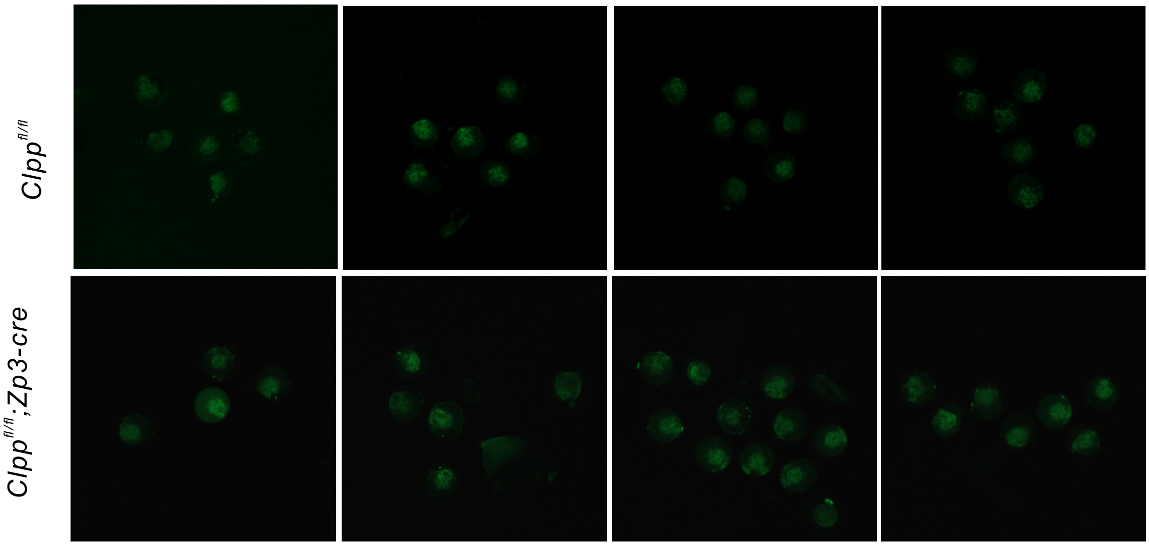

Fig 3-C Mito-traker staning

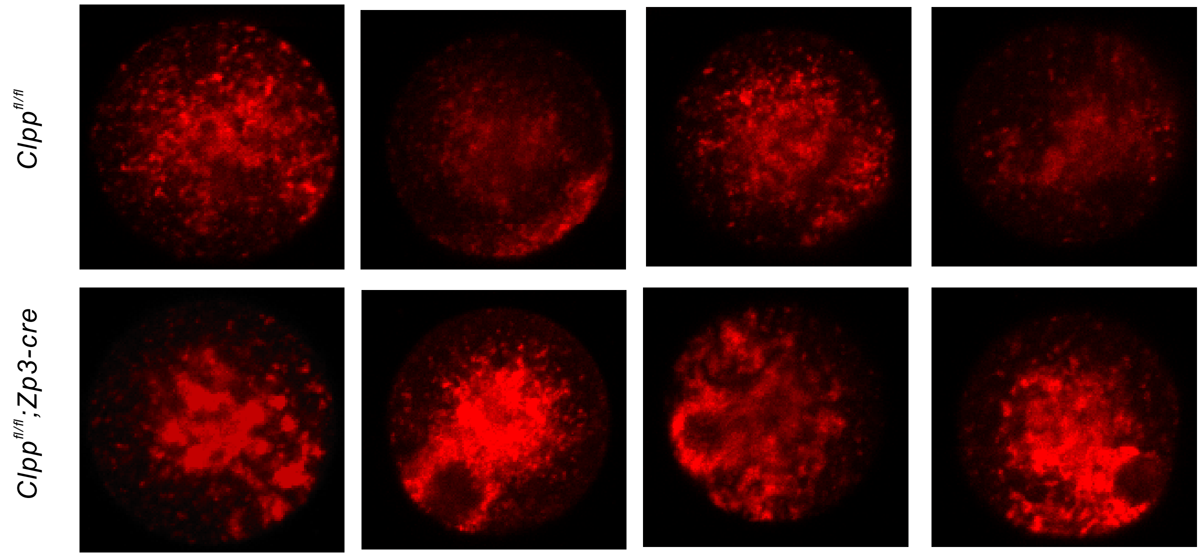

Fig 4-B Parthenogenetic activation image

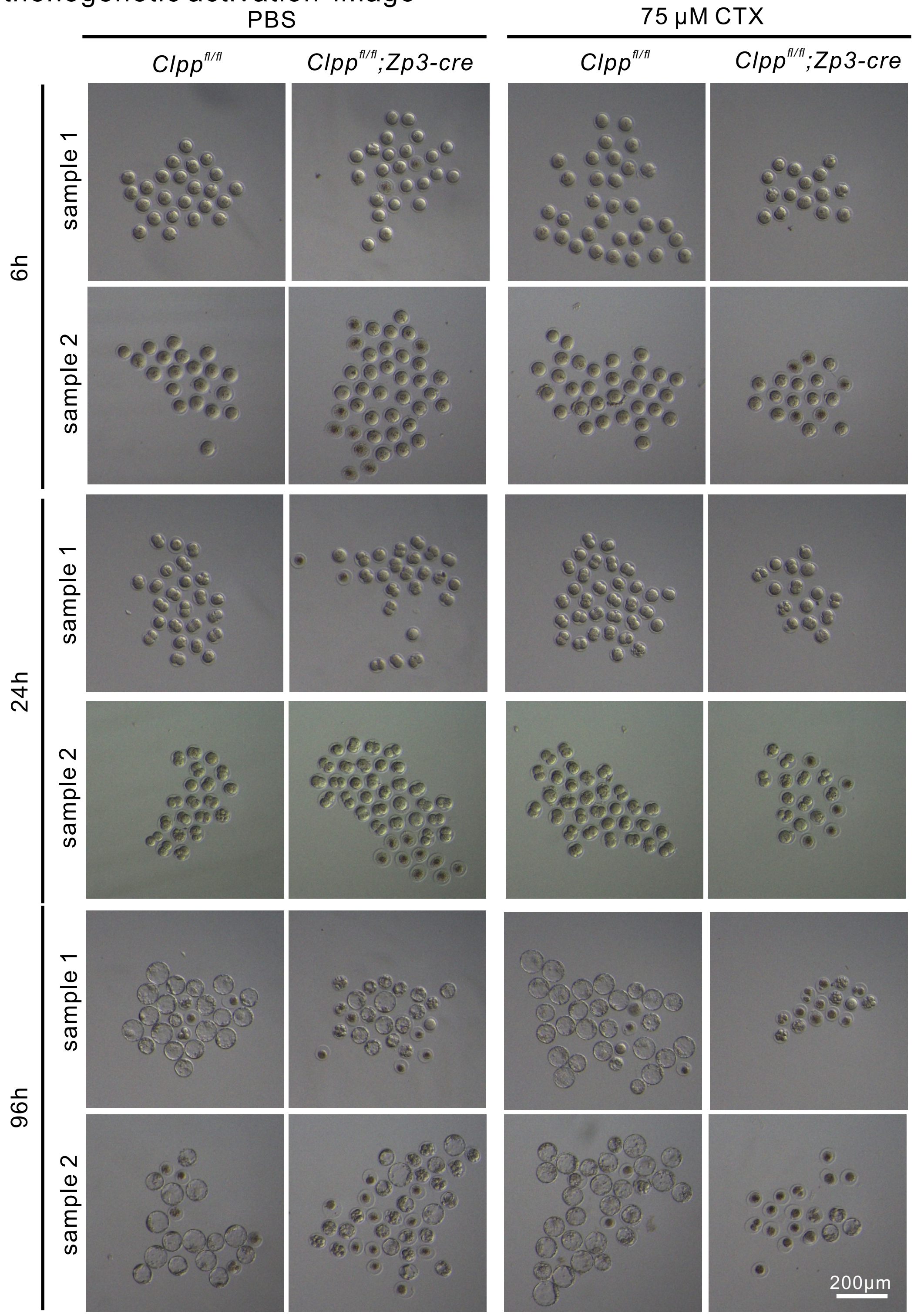

Fig 4-E Histological observation

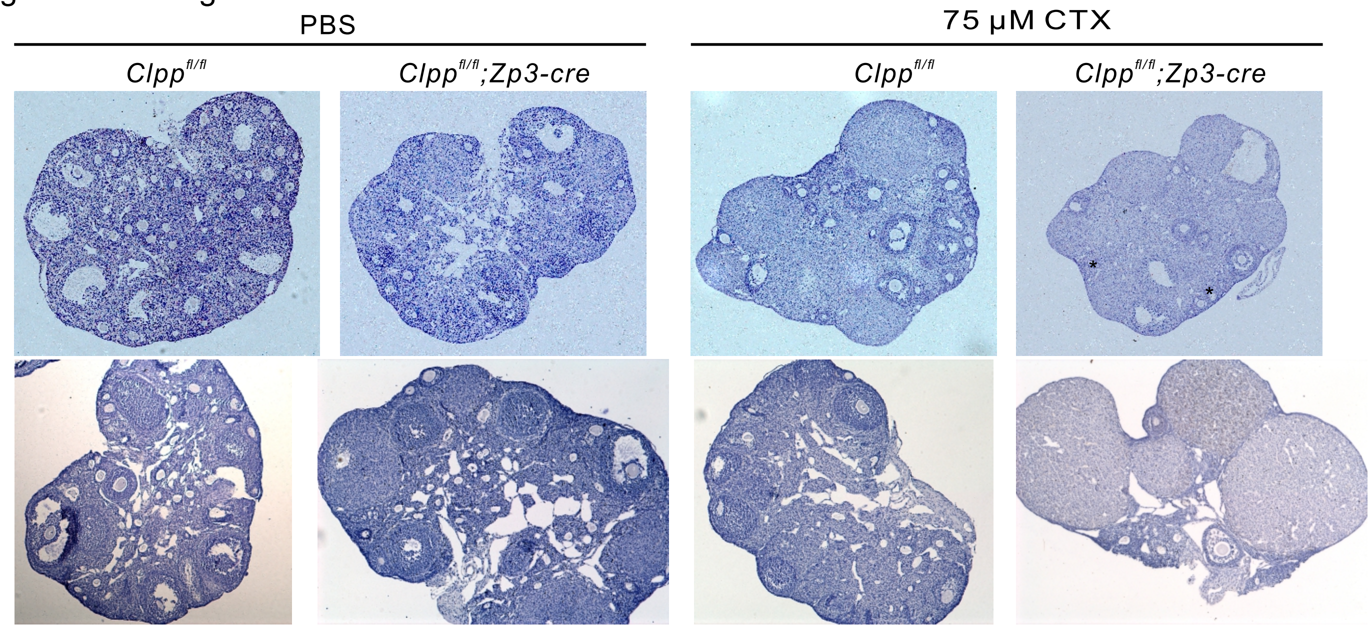

Fig 5-A Jc-1 staning

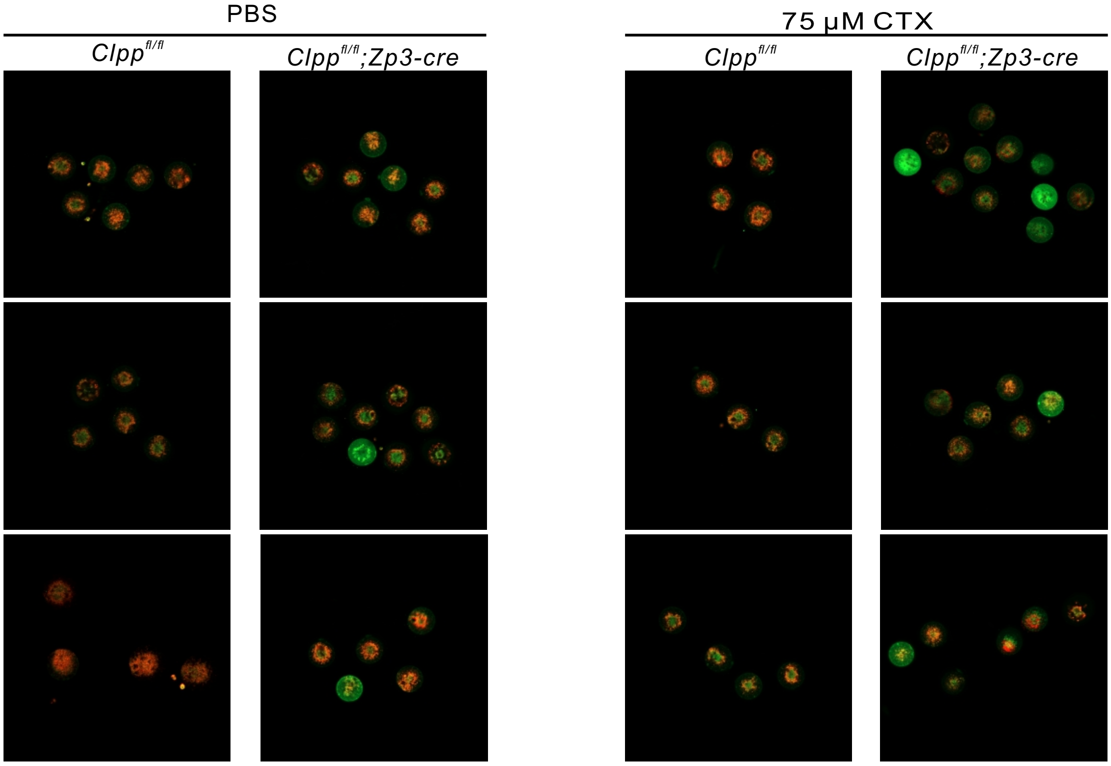

Fig 5-B ROS level staning

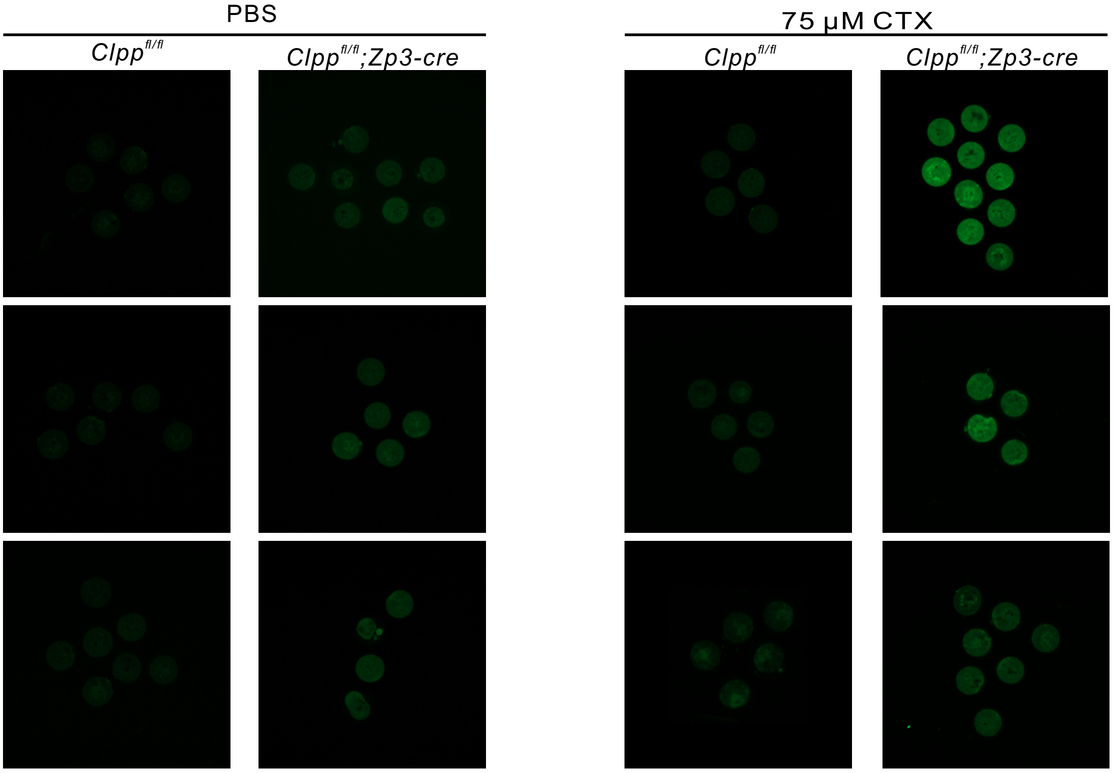

Fig 2-G Chromosomal spread image

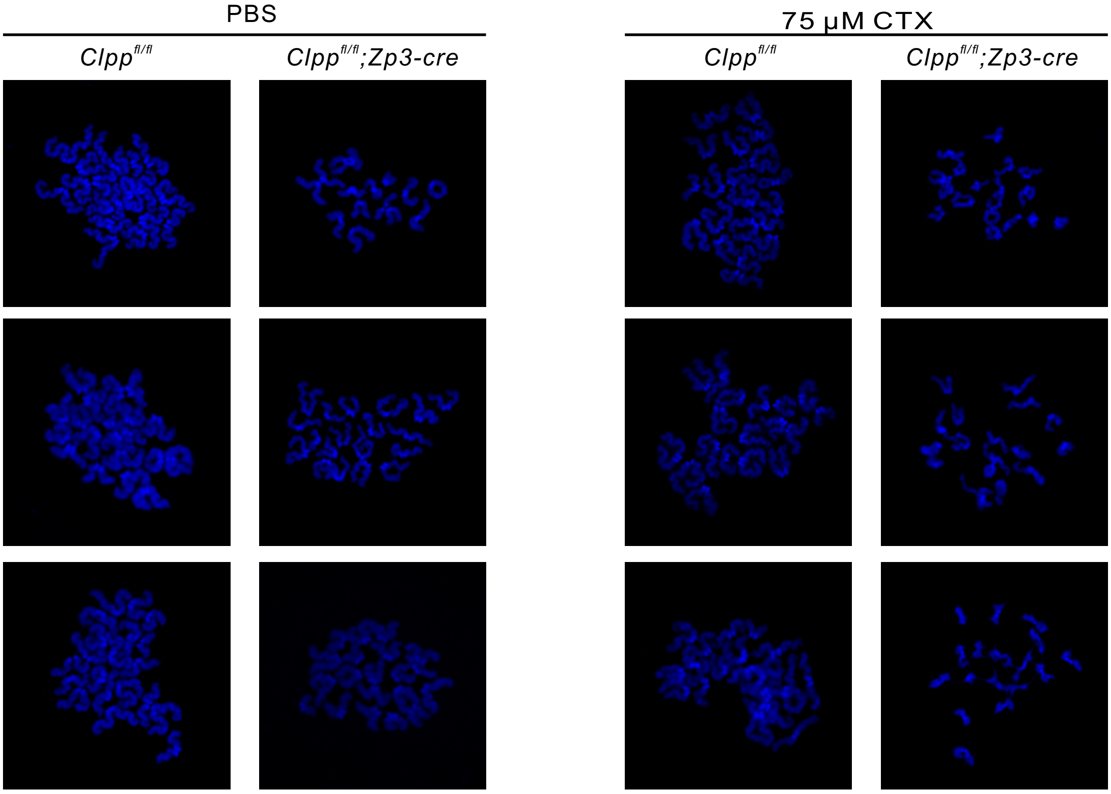

Fig 5-H Mito-traker staning

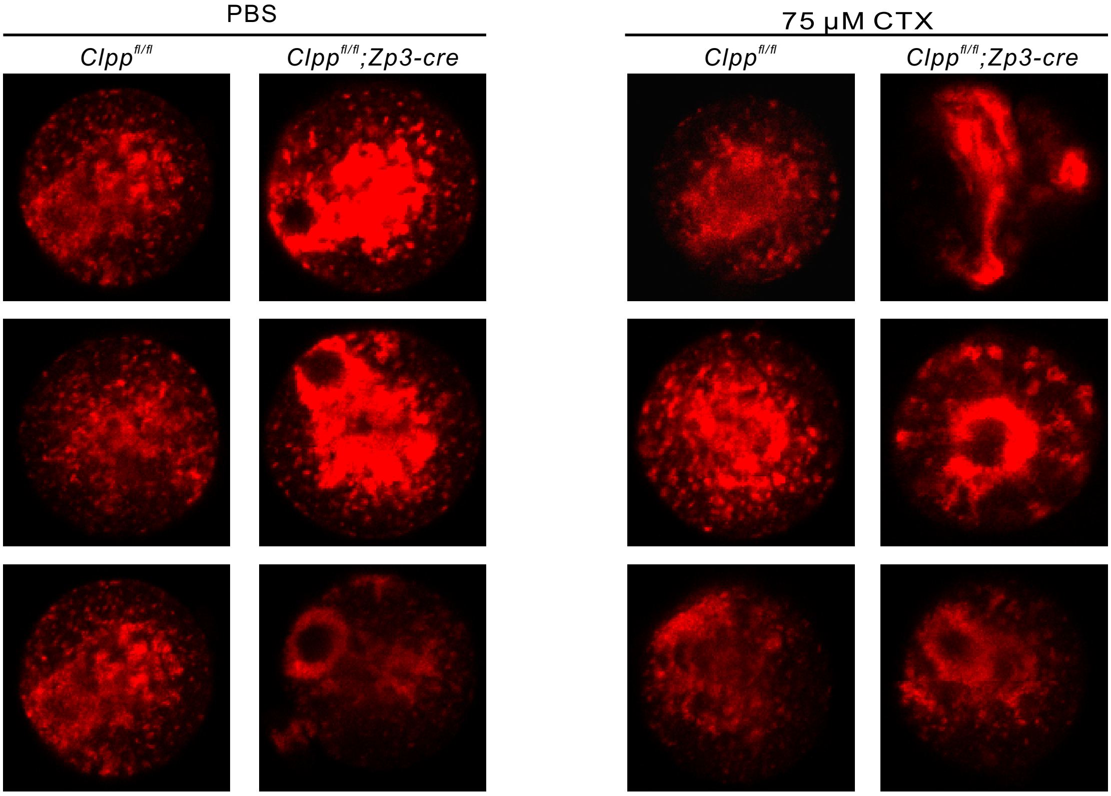

Supplement: Supplementary file 1 [file DataSheet_1.pdf]
