# Supplementary material for: Mitochondrial stress response gene Clpp deficiency impairs oocyte competence and deteriorate cyclophosphamide-induced ovarian damage in young mice
Source: Front Endocrinol (Lausanne). 2023 Mar 24;14:1122012. doi: 10.3389/fendo.2023.1122012 (PMC10081448; doi:10.3389/fendo.2023.1122012)
Supplement: Supplementary file 8 [file Table_7.docx]

Supplementary Material

# Supplementary Tables S1

The sequence list of gRNAs and primers

| Gene names | Sequence | Accession codes |
| --- | --- | --- |
| gRNA1(matching reverse strand of gene) | AAATGTCCTGTCAGCTACATGGG |  |
| gRNA2(matching reverse strand of gene) | CATAGAACCTATGTCGGGCCAGG |  |
| *Clpp*-Het | Forward primer 5’-TCTTACCATAGTCTGCTGTTGTCA-3’  Reverse primer: 5’-CTACACCTGGTCTGGTTTAGGAAC-3’ | OX390160.1 |
| *Clpp*-cKO | Forward primer: 5’-TCTTACCATAGTCTGCTGTTGTCA-3’  Reverse primer: 5’-GGAACTTTTGTTTTGTGACTCTGG-3’ | OX389811.1 |
| *Zp3*-Cre | Forward primer: 5′-CAGATGAGGTTTGAGGCCACAG-3’  Reverse primer: 5′-GCGAACATCTTCAGGTTCTGC-3’ | JN964226.1 |
| *Clpp* | Forward primer: 5′-GCCTTGCCGTGCATTTCTC-3’  Reverse primer: 5′-CTCCACCACTATGGGGATGA-3’ | XM_021218029.2 |
| *Cox3* | Forward primer: 5′-TAACCCTTGGCCTACTCACC-3’  Reverse primer: 5′-AATAGGAGTGTGGTGGCCTTG-3’ | LC062001.1 |
| *Sdhb* | Forward primer: 5′-AATTTGCCATTTACCGATGGGA-3’  Reverse primer: 5′-AGCATCCAACACCATAGGTCC-3’ | XM_034503832.1 |
| *Uqcrc2* | Forward primer: 5′-AAAGTTGCCCCGAAGGTTAAA-3’  Reverse primer: 5′-AAAGTTGCCCCGAAGGTTAAA-3’ | NM_025899.1 |
| *Atp5a1* | Forward primer: 5′-TCTCCATGCCTCTAACACTCG-3’  Reverse primer: 5′-CCAGGTCAACAGACGTGTCAG-3’ | XM_032885388.1 |
| *Ndufv1* | Forward primer: 5′-TTTCTCGGCGGGTTGGTTC-3’  Reverse primer: 5′-GGTTGGTAAAGATCCGGTCTTC-3’ | NM_133666.3 |
| *Cox1* | Forward primer: 5′-GTGCTGGGGCAGTGCTGGAG-3’  Reverse primer: 5′-TGGGGCCTGAGTAGCCCGTG-3’ | NM_008969.4 |
| *β-actin* | Forward primer: 5′-ATGACCCAAGCCGAGAAGG-3’  Reverse primer: 5′-CGGCCAAGTCTTAGAGTTGTTG-3’ | NM_027493.3 |

# Supplementary Tables S2

The list of Antibody

| Primary antibodies | Host | Dilution rate | Catalog No. | Manufacturer | Country |
| --- | --- | --- | --- | --- | --- |
| Anti-ClpP | Rabbit | 1:1000 | ab124822 | Abcam | Cambridge, UK |
| Alexa Fluor 488 conjugated-anti-α-tubulin antibody | Mouse | 1:200 | 16-232 | Millipore, Billerica | MA, USA |
| Anti-Beta-Actin | Rabbit | 1:1000 | ab227387 | Abcam | Cambridge, UK |
|  | | | | | |
| Secondary antibodies | Host | Dilution rate | Cat | Manufacturer | Country |
| HRP conjugated goat anti-rabbit IgG (H+L) cross-adsorbed antibody | Goat | 1:5000 | G-21234 | Invitrogen | MA, USA |

# Supplementary Tables S3.

The list of Main Reagent

| The full name of the reagent | Cat | Manufacturer | Country |
| --- | --- | --- | --- |
| Cyclophosphamide | HY-17420 | Mce | NJ, USA |
| The Rapid Taq polymerase | P222-02 | Vazyme | Nanjing, China |
| PBS | C10010500BT | Thermo Fisher Scientific | MA, USA |
| Neutral balsam | G8590 | Solarbio | Beijing, China |
| PMSG | P9970-1000 | Solarbio | Beijing, China |
| hCG | NB1122 | NSHF | Ningbo, China |
| M2 medium | M7167 | Sigma | MO, USA |
| hyaluronidase | H3506 | Sigma | MO, USA |
| DAPI | G1012-10ML | Solarbio | Beijing, China |
| MitoTracker™ Red CMXRos | M7512 | Invitrogen | MA, USA |
| Qiagen plasmid isolation kit | 12145 | Qiagen | Hilden, Germany |
| Taq Pro Universal SYBR qPCR Master Mix | Q712-02 | Vazyme | Nanjing, China |
| carboxy-H2DCFDA | C-400 | Life Technologies | MA, USA |
| JC-1 | T3168 | Invitrogen | MA, USA |
| ATP Bioluminescent Somatic Cell Assay Kit | FLASC-1KT | Sigma | MO, USA |
| HiScript III RT SuperMix for qPCR (+gDNA wiper) kit | R323-01 | Vazyme | Nanjing, China |
| Triton X-100 | 85111 | Thermo Fisher Scientific | MA, USA |
| DTT | R0862 | Thermo Fisher Scientific | MA, USA |
